# Supplementary material for: Pathway Commons 2019 Update: integration, analysis and exploration of pathway data
Source: Nucleic Acids Res. 2019 Oct 24;48(D1):D489–97. doi: 10.1093/nar/gkz946 (PMC7145667; doi:10.1093/nar/gkz946)

SUPPLEMENTARY DATA

Table of Contents

Purpose of this document 1
Case Studies 1
  Example Usage of PC Web Apps 2
  Example Workflows 4
Description of Simple Interaction Format (SIF) 9
  Binary interaction patterns in a BioPAX model 9
    controls-state-change-of 9
      via direct control 10
      as a participant 10
      as a controlling input 11
      through controller small molecule 12
      through binding small molecule 13
      through degradation 14
    controls-phosphorylation-of 15
    controls-transport-of 16
    controls-expression-of 17
      through TemplateReaction 17
      through Conversion 18
    catalysis-precedes 18
    in-complex-with 19
    interacts-with 20
    neighbor-of 20
    consumption-controlled-by 21
    controls-production-of 22
    controls-transport-of-chemical 22
    chemical-affects 23
      through binding 23
      through a control 24
    reacts-with 24

used-to-produce 25
Example pathways using binary interactions 25
The Citric Acid Cycle 26
TCA cycle from wikipathways 26
Binary network from Pathway Commons 26
BMP signaling 27
Wiki pathways 28
Binary network from Pathway Commons 28
AMPK signaling 29
Wiki pathways 29
Binary network from Pathway Commons 29
ABC-mediated transport 31
Reactome 31
Binary network from Pathway Commons 31

Purpose of this document

This document contains “Case Studies” illustrating how some of the main software tools and libraries described in this report could be used to help solve research problems. This is followed by an in-depth description of a data output format in the section “Description of Simple Interaction Format (SIF)”. BioPAX can encode very detailed information about biological processes. Analysis of this data, however, can be complicated as one needs to consider a wide array of n-ary relationships, different states of entities and generic classes of molecules. We provide the data in an alternative format, called SIF, that is much simpler in structure and encodes some essential information in the BioPAX data. We search certain graph patterns in the BioPAX structure to detect SIF relations. This section describes those graph patterns and provides examples.

Case Studies

This section highlights a case study and example analysis workflows that make use of Pathway Commons apps and data.

Example Usage of PC Web Apps

Functional genomics aims to identify the genes and interactions that underlie an observed behavior or phenotype. One common experimental approach to this involves parallel perturbations of genes through use of RNA interference (RNAi), and more recently CRISPR technology, to identify gene candidates or 'hits' that modify the phenotype. The PC web apps provide a useful integrated system to support researchers in answering questions such as "What is known about this gene hit?", "Do any of these hits interact with one another?" and "How do these hits relate to the behavior I'm interested in?"

To illustrate how PC apps can provide this type of insight, we refer to an investigation by Miller et al. (1, 2) who introduced RNAi in patient-derived glioblastoma xenografts and identified 60 candidate genes that may contribute to cancerous growth. Pasting this list into the PC Search app returns relevant information including links to more information about recognized genes (figure below, top), PC apps (panels labelled "Interactions" and "Enrichment") and pathway hits.

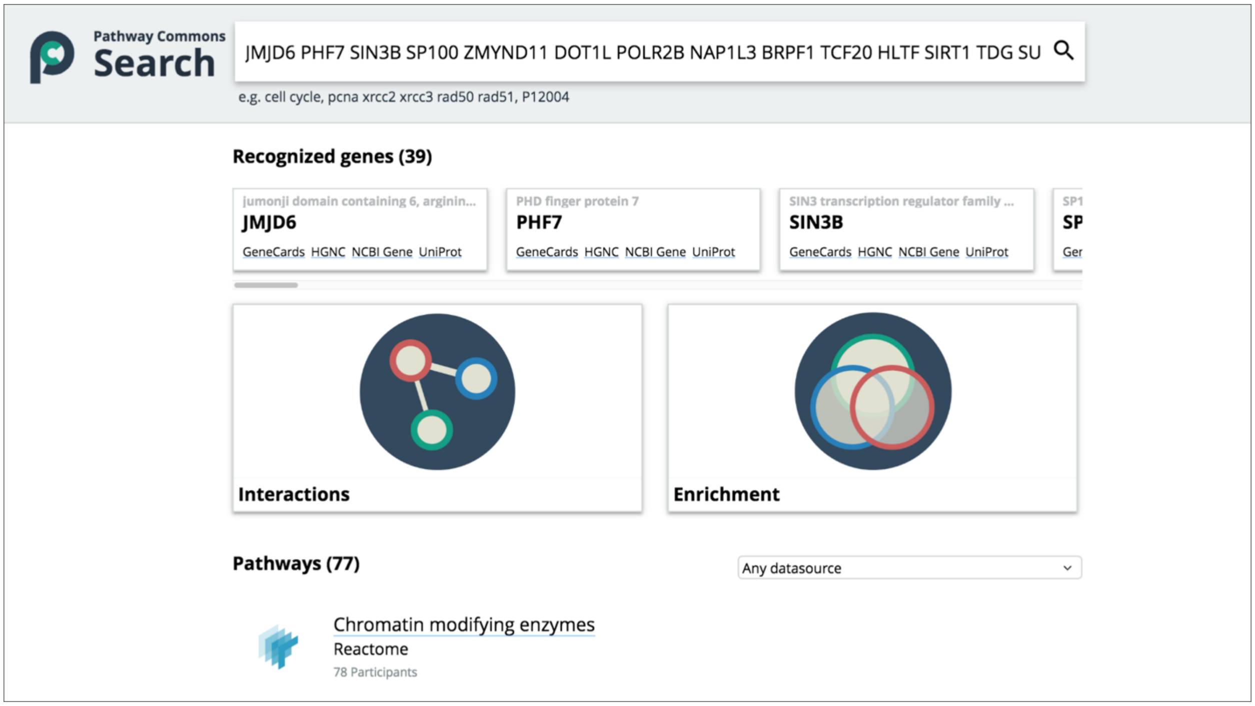

To gain insight into how these hits may influence glioblastoma growth, a first step would be to determine what pathways are enriched for these genes. The Enrichment app (figure above, right) analyzes gene inputs and displays pathways in the form of a network formatted as an "Enrichment Map", where nodes represent pathways (larger ones have more genes) and edges connect pathways with overlapping genes (thickness indicates extent of overlap).

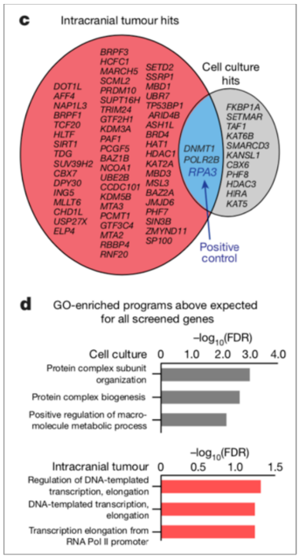

Figure 1
Miller *et al.* (2017) Nature 547

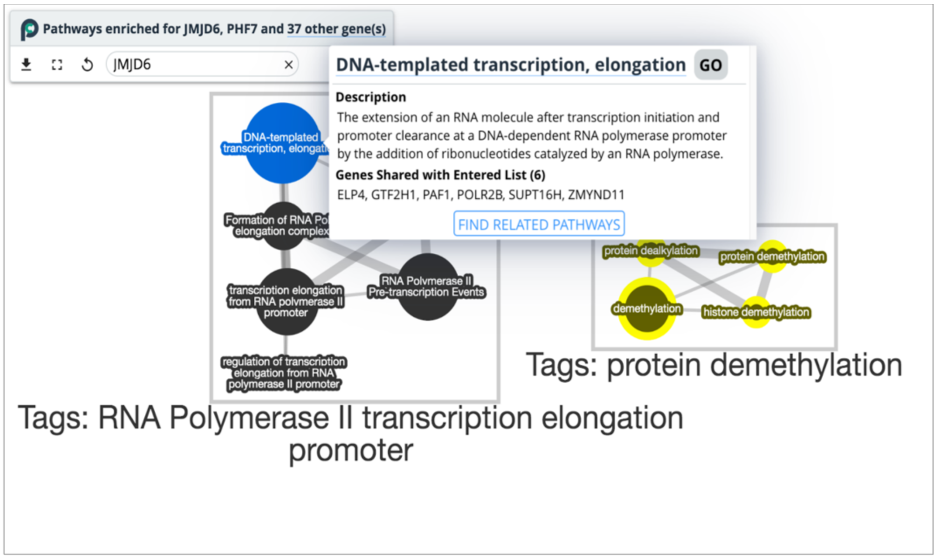

'Enrichment' App

Miller et al. used Gene Ontology analysis to discover that '... in vivo-specific hits were enriched for genes controlling transcriptional elongation (Fig. 1d and Extended Data Fig. 3c, d)'. In the above figure, we see that the pathway data in Miller et al. were presented as a bar chart (red bars, left), which does not provide any information about if or how the various pathways are related. In contrast, the Enrichment app (right) automatically clusters similar pathways and summarizes then with tags of text drawn from the constituent pathway labels, for instance, 'RNA Polymerase II transcription elongation promoter'. Moreover, the Enrichment app is interactive and explorable, enabling us to locate genes of interest within the pathway nodes (e.g. yellow highlights pathways containing the gene 'JMJD6') and pathway tooltips display additional information (e.g. 'DNA-templated transcription, elongation'), links to the original data providers webpage and even a one-click option to 'FIND RELATED PATHWAYS' by querying PC Search with the pathway name.

The remainder of the study by Miller et al. focused on the gene jumonji C-domain-containing protein 6 (JMJD6) because it was found to be a key driver of gene expression required for growth in glioblastoma cells. Indeed, JMJD6 knockout cells generated by CRISPR-Cas9 technology resulted in poor glioblastoma growth, providing preclinical support for therapeutically targeting JMJD6. In this context, a researcher might be interested in more information to hypothesize about topics like 'What else does JMJD6 interact with in order to modulate transcriptional pausing?' and 'Are there other genes that JMJD6 interacts with that could represent viable therapeutic targets?'.

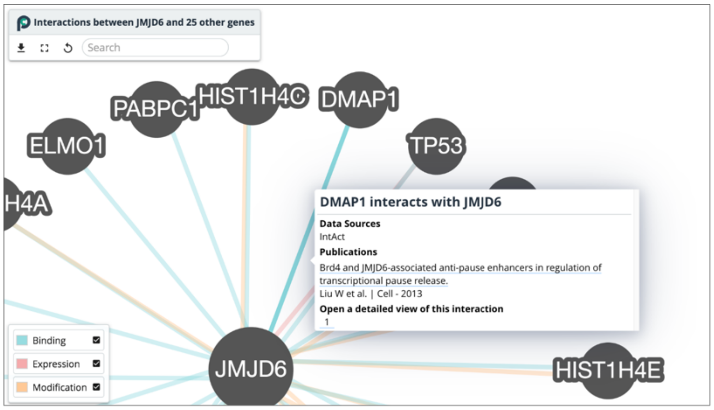

'Interactions' App

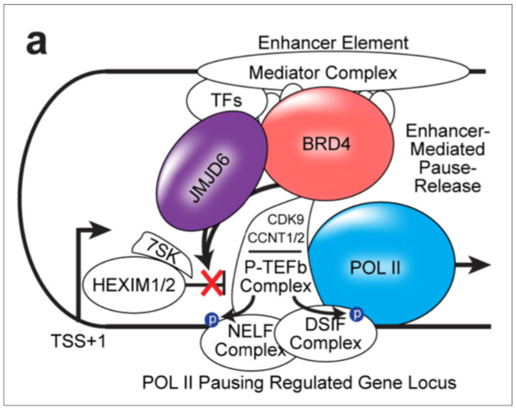

(Extended) Figure 8
Miller *et al.* (2017) Nature 547

To provide clues to these questions, a user can query PC Search with JMJD6. In this case, the Interactions app offers a visual network of known relationships as shown in the figure above (left). For instance, an interaction with DMAP1 is displayed and a tooltip refers to a published study detailing the post-translational modifications brought about by JMJD6 and BRD4 in order to reduce RNA polymerase II pausing at proximal promoters (3). Indeed, Miller et al. used this same study to create a figure (right) that depicts how a subset of the candidates identified in the RNAi screen interact and cooperate.

Altogether, this example illustrates how PC apps could be used to aid researchers in interpreting genomics data experiments, finding out more information about genes and generating additional hypotheses.

Example Workflows

In addition to this case study, we summarize four pathway analysis workflow examples that make use of Pathway Commons data and have been used in multiple papers.

- **Tool:** EnrichmentMap Cytoscape app for Gene Set Enrichment Analysis (GSEA) pathway enrichment analysis result visualization.
    - **Question/Goal:** Which biological processes are active/altered in my experiment?
    - **Method Description:** Statistical over-representation and removal of gene set redundancy.
    - **Example:** The EnrichmentMap app summarizes the results of pathway enrichment analysis. The figure below shows an EnrichmentMap for gene expression changes after estrogen treatment of MCF7 breast cancer cells at 24 hours. Each node represents a pathway. Upregulated pathways (red) include those related to DNA metabolism, cell cycle, transcription/translation, while processes such as lipid transport are downregulated estrogen treated cells versus untreated cells.
    - **Citation:** (4)

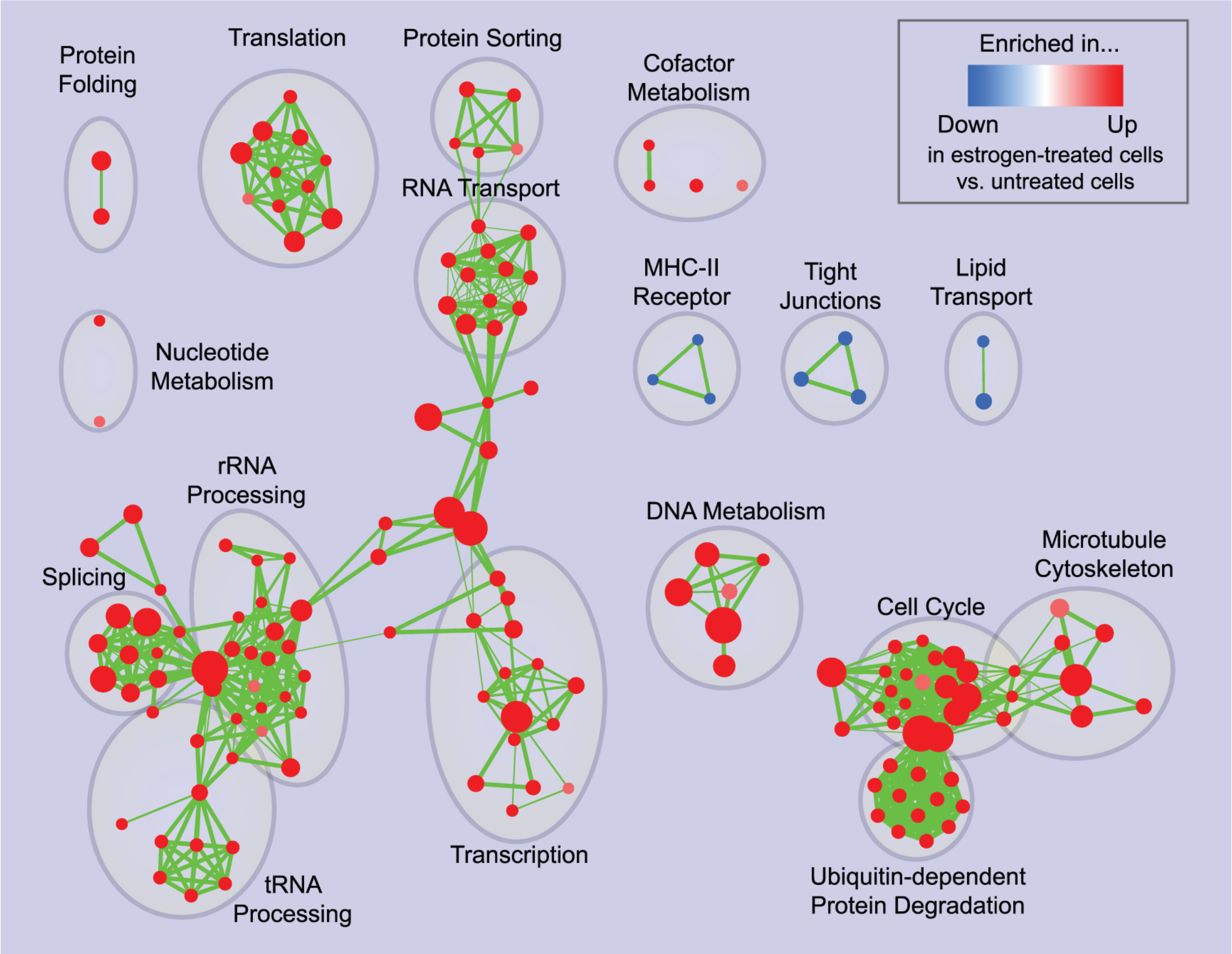

- **Tool:** NetBox
    - **Question/Goal:** Infer modules (pathways) from data and identify novel cancer driver candidates
    - **Method Description:** Clustering by network paths & statistical test
    - **Example:** Using alteration information in the TCGA glioblastoma data set, NetBox is able to identify network modules using Pathway Commons data to suggest altered processes. Additionally, NetBox nominates potential candidate cancer-driving genes (i.e., linker genes) via analysis of the network topology of the input list of altered genes (see figure below).
    - **Citation:** (5)

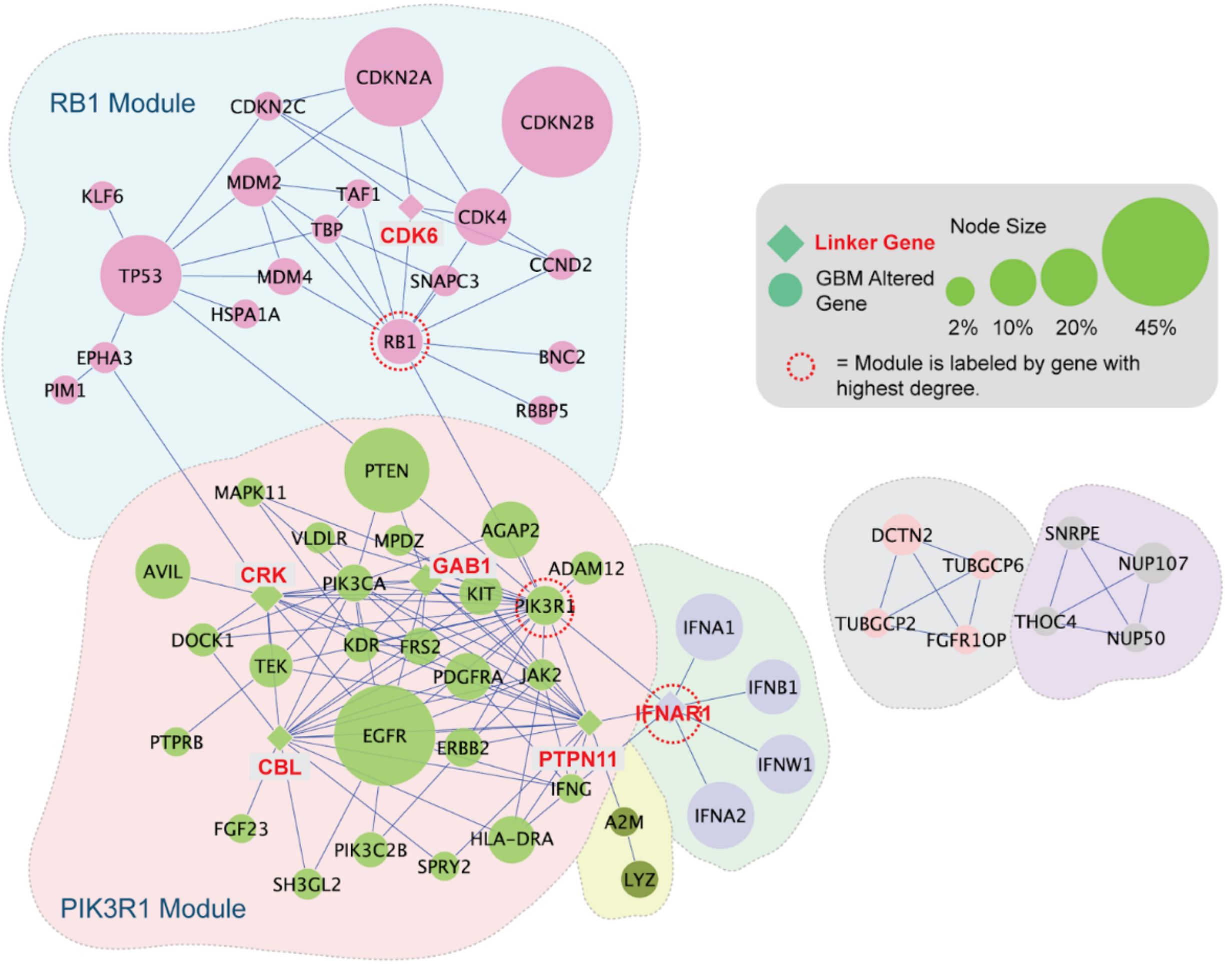

- **Tool:** CausalPath
    - **Question/Goal:** Find molecular relations that can causally explain concordant changes in a given set of molecular measurements.
    - **Method Description:** Graphical pattern search on BioPAX models to detect potential causal relations between observables in molecular profiles, then select the subset of the relations that can causally explain the coordinated behavior in pairs of given measurements.
    - **Example:** CausalPath can be used to identify signaling cascades via phosphorylation change upon a perturbation. Below is an example result network for phosphoproteomic profiles from a platelet activation experiment where ADP was used as the activating agent. CausalPath reconstructs several known signaling paths in platelet activation (such as MAPK signaling), and suggests several new relations that it knows from other biological contexts but also can explain this dataset (such as MAPK signaling causing RTN4 phosphorylation).
    - **Citation:** (6)

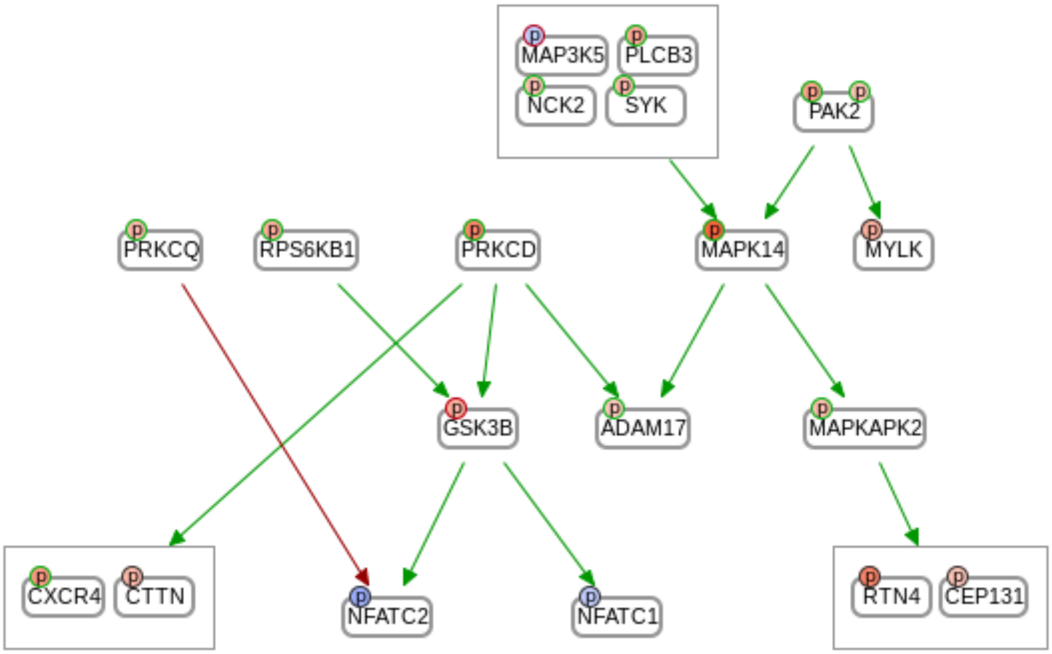

- **Tool:** Mutex
    - **Question/Goal:** Discover functional mutations in a disease cohort.
    - **Method Description:** Test mutual exclusivity of alterations of gene groups that control a common downstream signaling target.
    - **Example:** Groups of genes with mutually exclusive alterations in breast invasive carcinoma are extracted using the mutex method along with interactions of these extracted groups. Note: the color red in figure indicates the percent alteration across samples.
    - **Citation:** (7)

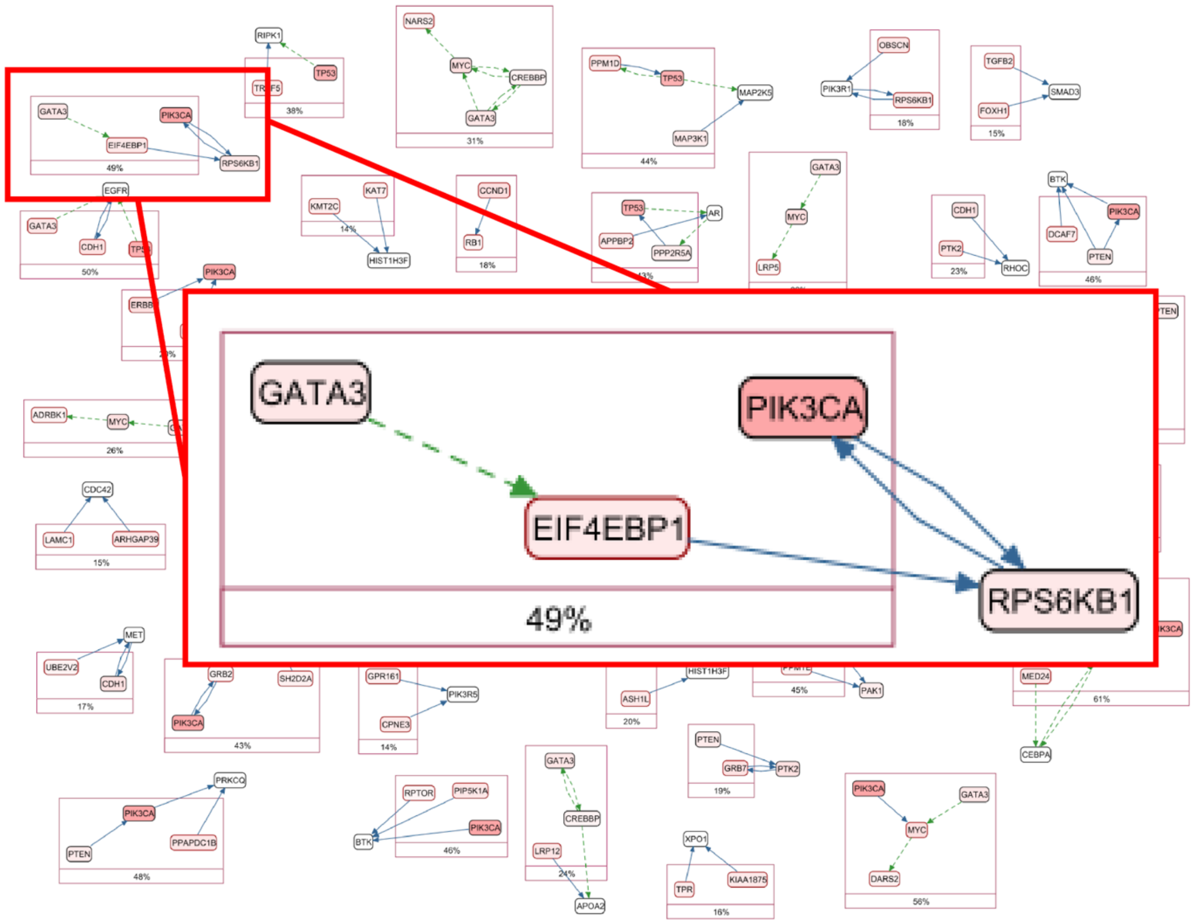

1. Northcott,P.A. (2017) Cancer: Keeping it real to kill glioblastoma. *Nature*, **547**, 291–292.
2. Miller,T.E., Liau,B.B., Wallace,L.C., Morton,A.R., Xie,Q., Dixit,D., Factor,D.C., Kim,L.J.Y., Morrow,J.J., Wu,Q., *et al.* (2017) Transcription elongation factors represent in vivo cancer dependencies in glioblastoma. *Nature*, **547**, 355–359.
3. Liu,W., Ma,Q., Wong,K., Li,W., Ohgi,K., Zhang,J., Aggarwal,A. and Rosenfeld,M.G. (2013) Brd4 and JMJD6-associated anti-pause enhancers in regulation of transcriptional pause release. *Cell*, **155**, 1581–1595.
4. Merico,D., Isserlin,R., Stueker,O., Emili,A. and Bader,G.D. (2010) Enrichment map: a network-based method for gene-set enrichment visualization and interpretation. *PloS One*, **5**, e13984.
5. Cerami,E., Demir,E., Schultz,N., Taylor,B.S. and Sander,C. (2010) Automated network analysis identifies core pathways in glioblastoma. *PloS One*, **5**, e8918.
6. Babur,Ö., Luna,A., Korkut,A., Durupinar,F., Siper,M.C., Dogrusoz,U., Aslan,J.E., Sander,C. and Demir,E. (2018) Causal interactions from proteomic profiles: molecular data meets pathway knowledge Systems Biology. BioRxiv p.258855
7. Babur,Ö., Gönen,M., Aksoy,B.A., Schultz,N., Ciriello,G., Sander,C. and Demir,E. (2015) Systematic identification of cancer driving signaling pathways based on mutual exclusivity of genomic alterations. *Genome Biol.*, **16**, 45.

Description of Simple Interaction Format (SIF)

Binary interaction patterns in a BioPAX model

We define the below binary interaction types for capturing certain relations in a BioPAX model:

controls-state-change-of
controls-phosphorylation-of
controls-transport-of
controls-expression-of
catalysis-precedes
in-complex-with
interacts-with
consumption-controlled-by
controls-production-of
controls-transport-of-chemical
chemical-affects
reacts-with
used-to-produce

An interaction type can be captured with more than one pattern in a BioPAX model. For instance we use 6 different patterns to represent the controls-state-change-of relation.

In the current proposal, an interaction type can have either a protein or a small molecule as source or target. For a specific interaction type, the type of the source or target is fixed. For instance the first 8 interaction types in the above list are always between proteins. The next 4 interaction types are always between a protein and a small molecule. The last 2 are always between small molecules.

Proteins are identified with HGNC gene symbols. Even though a gene can produce more than one protein, and UniProt IDs are better suited for identifying proteins, we sacrifice this resolution to create readable node names. We identify small molecules with the display names of their SmallMoleculeReference.

controls-state-change-of

This relation defines directed interactions between proteins. It means the first protein has an effect on an event that changes the state of the second protein. Many signaling relations that are transmitted through protein modifications are captured with this relation.

This relation has 2 sub-relations: controls-phosphorylation-of and controls-transport-of.

via direct control

The first protein is a controller of an interaction that modifies the second protein post-translationally. The controller protein is also not involved in the reaction as a participant.

Example: A complex of Ephrin transfers GDP to HRAS, and a complex of AR transfers GTP to HRAS.

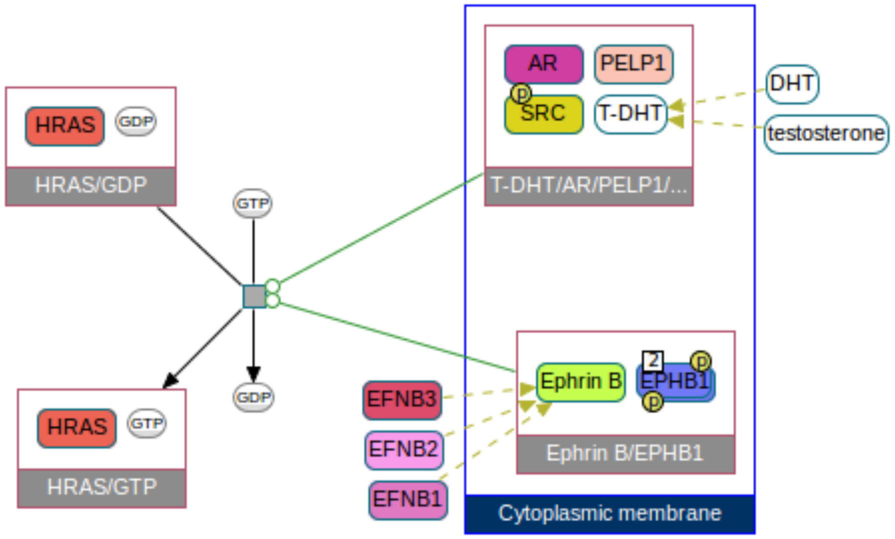

Using the data above, we generate the controls-state-change-of relations below.

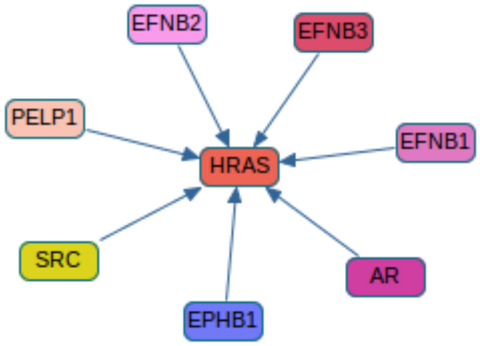

as a participant

Sometimes Reactome prefers an odd way of showing controllers. They make the controller both input and output using the same PhysicalEntity. This pattern captures it.

Example: An Interferon-JAK-Stat complex phosphorylates STAT1.

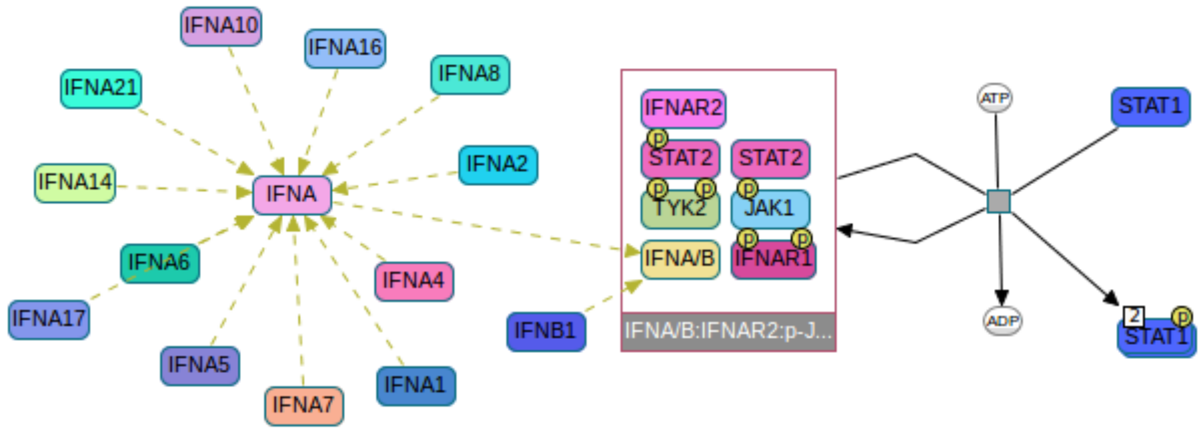

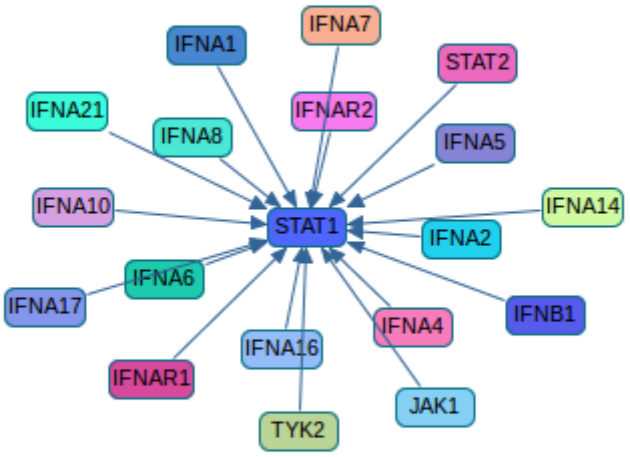

as a controlling input

This is another Reactome pattern for controls-state-change-of. The controller molecule is also an input to the conversion; and it is also associated with output with the same non-generic physical entity. The affected protein is represented with different simple physical entities on both sides of the reaction, where each simple physical entity is associated with only one side.

Example: SYK is phosphorylated by a complex containing LCK and FYN. SYK is also a member of this complex.

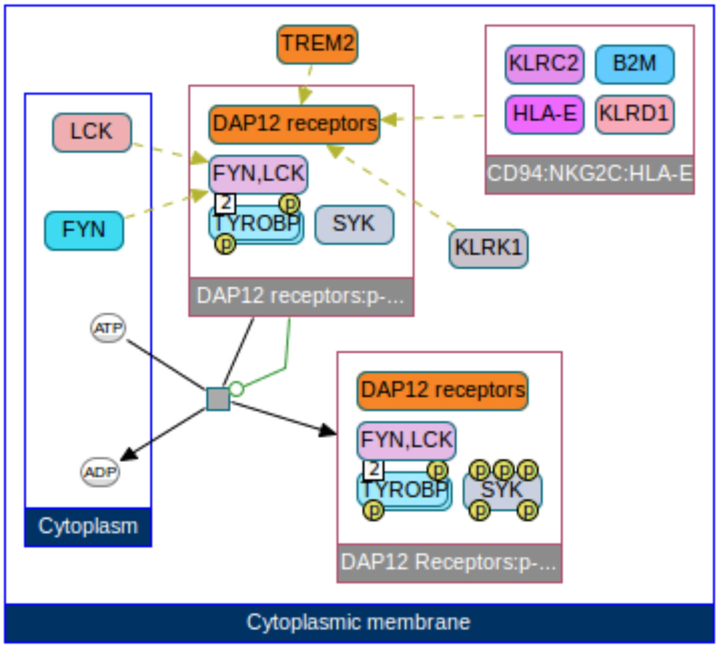

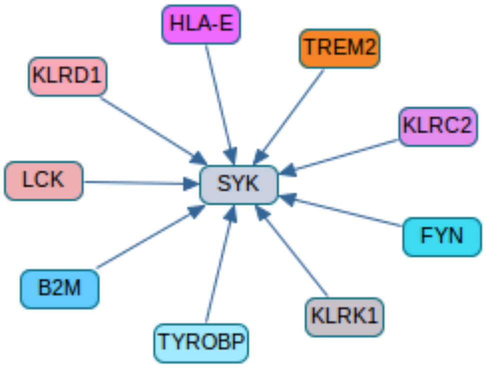

through controller small molecule

First protein controls a conversion that produces a small molecule. This small molecule controls a conversion that modifies the second protein. The first reaction should be changing the concentration of the linker small molecule. This means the linker small molecule should not be a blacklisted ubiquitous molecule.

<u>Example:</u> Activated SMPD2 catalyzes a reaction that turns sphingomyelin into ceramide. Ceramide activates another reaction that activates JNK family proteins by phosphorylation.

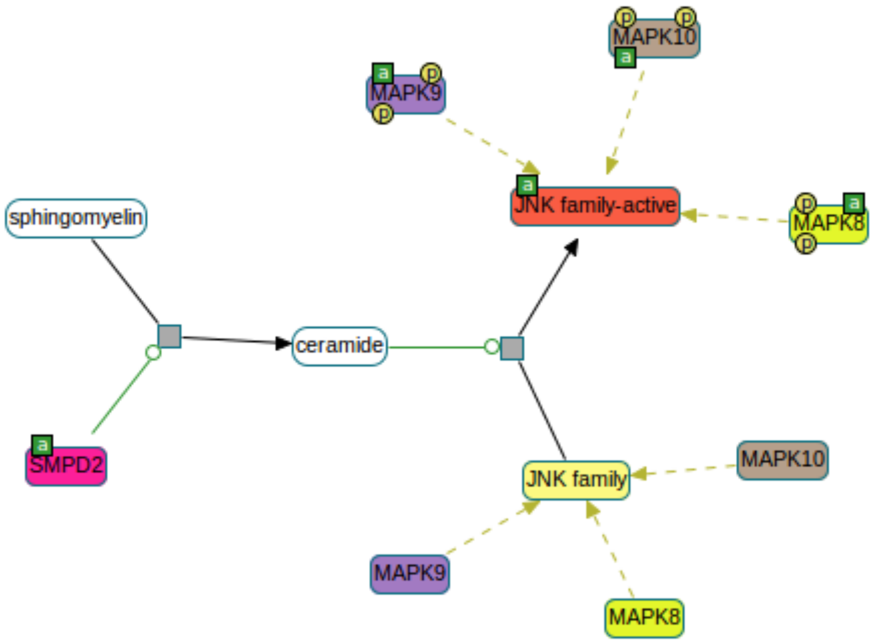

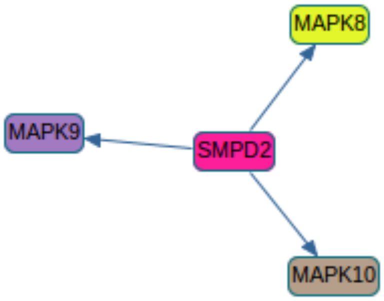

through binding small molecule

First protein controls a reaction that produces a small molecule. The small molecule forms a complex with the second protein. The first reaction should be changing the concentration of the linker small molecule.

<u>Example:</u> The PIK3CA complex catalyses production of PIP3. PIP3 binds to an AKT1 complex.

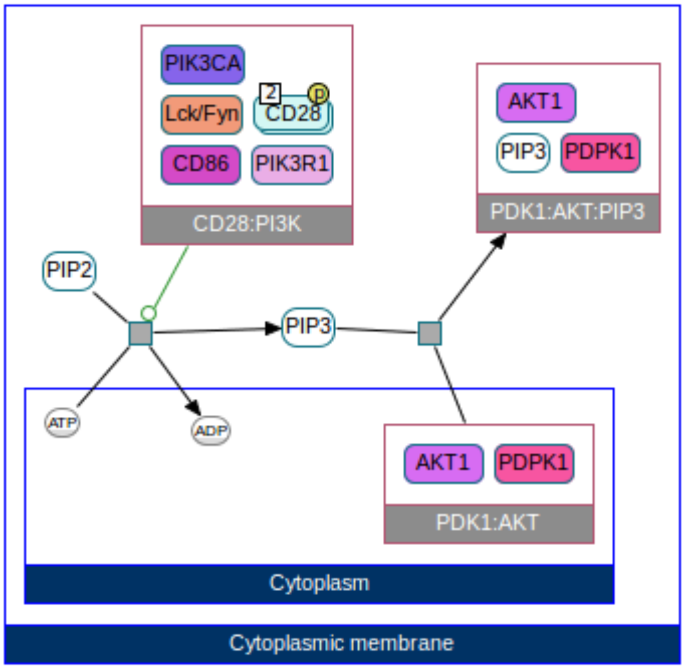

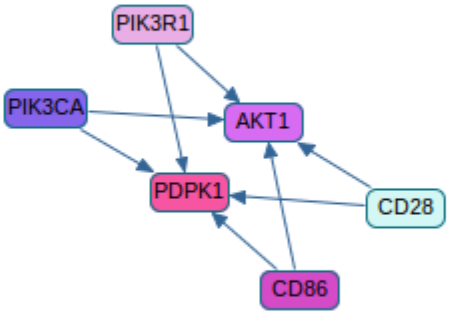

through degradation
First protein is controlling a reaction that degrades the second protein.

Example: Smurf proteins control degradation of SMADs.

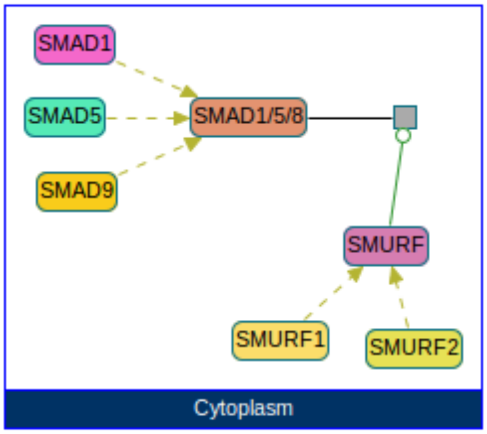

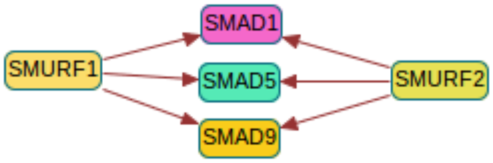

controls-phosphorylation-of
This is directed relation between proteins in which the first protein changes the phosphorylation status of the second protein. This is a sub-relation of controls-state-change-of. That means every controls-phosphorylation-of relation is also a controls-state-change-of relation. This relation can be useful for researchers studying phospho-proteomics.

<u>Example:</u> RAF phosphorylates MEK.

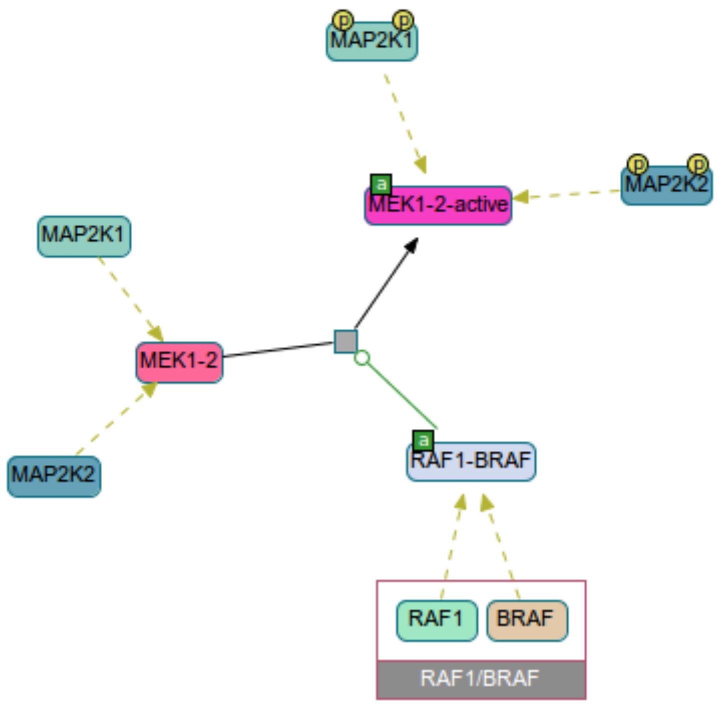

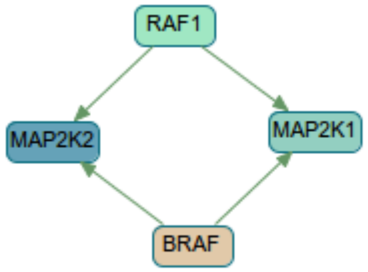

controls-transport-of

This is a directed relation between proteins in which the first protein controls a reaction that changes the cellular location of the second protein. Changing cellular location from null to something and from something to null does not qualify as transportation. It has to be between two non-null values. This relation type is a sub-type of controls-state-change-of. That means every controls-transport-of relation is also a controls-state-change-of relation. This relation can be useful for researchers studying cell trafficking.

Example: PTPN11 makes GRB2 dissociate from a membrane bound complex, hence, changes its location to cytoplasm.

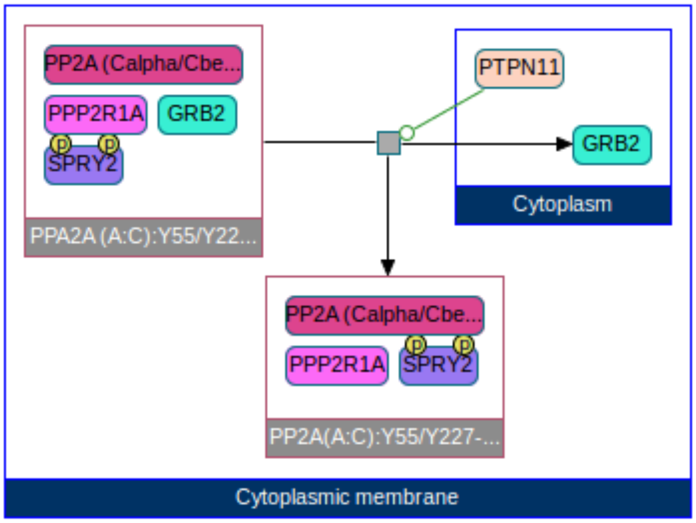

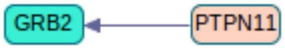

controls-expression-of

Defines a directed relation between two proteins where the first protein controls expression of the second protein. This relation can be useful for relating an alteration of a gene to another gene's expression change.

through TemplateReaction

The right way of modeling an expression in BioPAX is using a TemplateReaction. This pattern is composed of a controller, the controlled template reaction and the product.

Example: A complex of CREBBP activates expression of VEGFA.

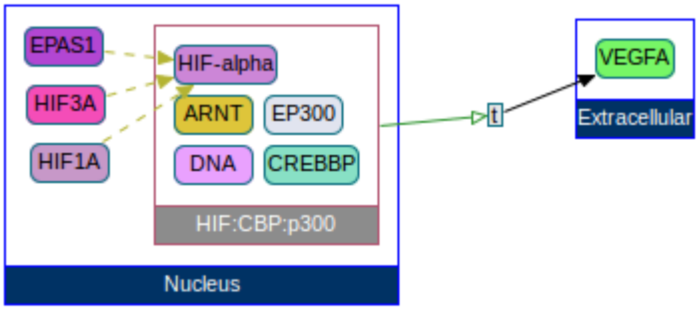

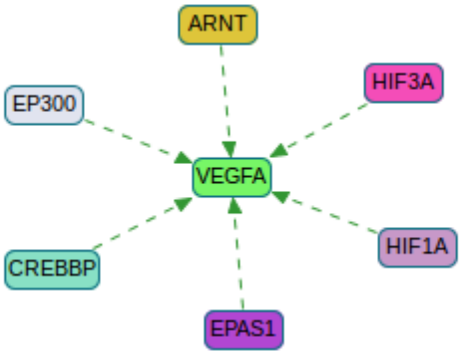

through Conversion

Some databases are still using a Conversion to model expression (NCI). These are conversions with only one right-participant, and no left-participant. This second pattern captures those expressions modeled in wrong way.

Example: RB1 bound TP53 inhibits TERT expression.

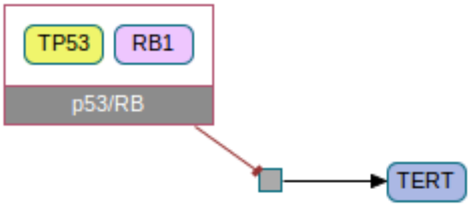

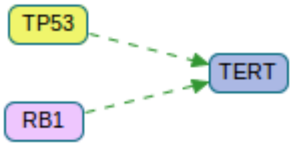

catalysis-precedes

Defines directed relations between two proteins that control consecutive reactions. The first protein controls a reaction whose output molecule is input to another reaction. This other reaction is controlled by the second protein. Both reactions are capable of changing concentration of the linker small molecule. The reactions cannot be identical or reverse of each other. Unshared participants at the non-facing sides of two reactions should contain at least one non-ubiquitous small molecule.

This relation can be useful for recovering metabolic cycles in the data.

Example: CYP2J2 turns arachidonate into epoxide, and epoxide hydrolyases turn the epoxide into a diol.

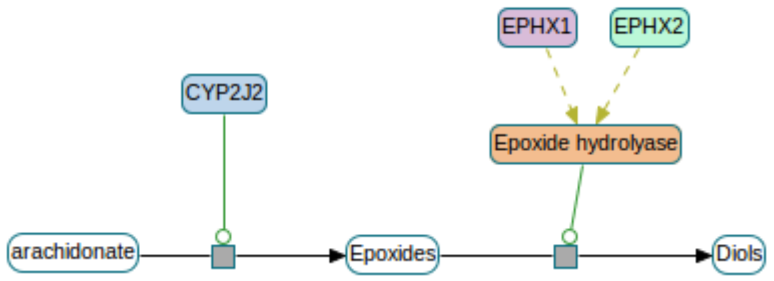

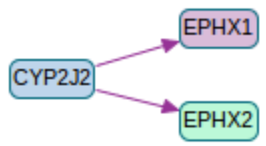

in-complex-with

Two proteins appear as components of the same complex. This excludes the pairs of components that cannot be members of the complex at the same time. In an ideal world, this undirected relation should reproduce the whole PPI network.

Example: WWTR1 and YAP1, separately, forms complexes with members of TEAD family. Notice that this relation is not defined between members of TEAD proteins.

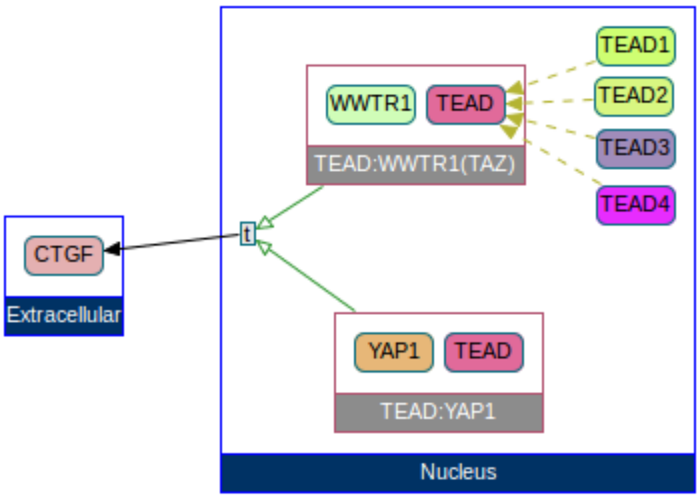

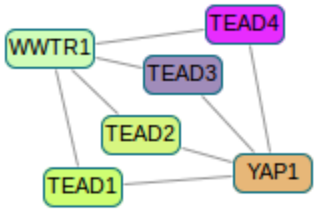

interacts-with

This is an undirected relation between participant proteins of a MolecularInteraction. This relation is for capturing protein interaction data (or data at the same level of simplicity), like data from HPRD.

neighbor-of

Two proteins are related to the same interaction (any kind), through being inputs, outputs, or controllers. This relation is useful when we need to get a list of related proteins, but don't care about the nature of the relation, and all we need is a vague neighborhood. This pattern tends to generate a lot of edges, hence, even small networks look like a hairball.

Relation to first version: This is a new interaction type. It resembles the previous INTERACTS_WITH, however this one also includes controllers of the interactions.

Example: A cyclin/CDK complex makes RB to dissociate from E2F complex. Resulting binary network is a clique with all 12 non-generic proteins involved in the process.

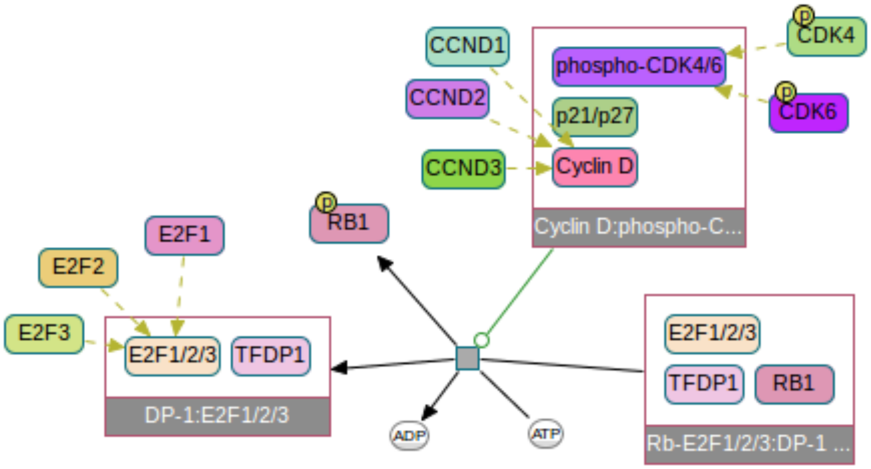

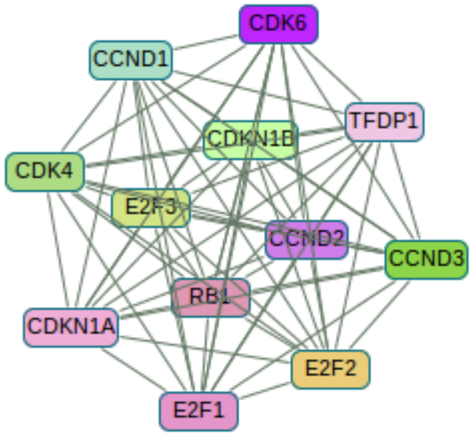

consumption-controlled-by

This is a directed relation from a small molecule to a protein. The protein is controlling the consumption of the small molecule. The reaction is supposed to change the concentration of the small molecule. The consumed small molecule (input) cannot also be an output. This relation can be useful for studying metabolic events.

Example: Below is part of the TCA cycle, where FH catalyses Fumaric acid and L-Malate, and MDH2 catalyses L-Malate and Oxaloacetate.

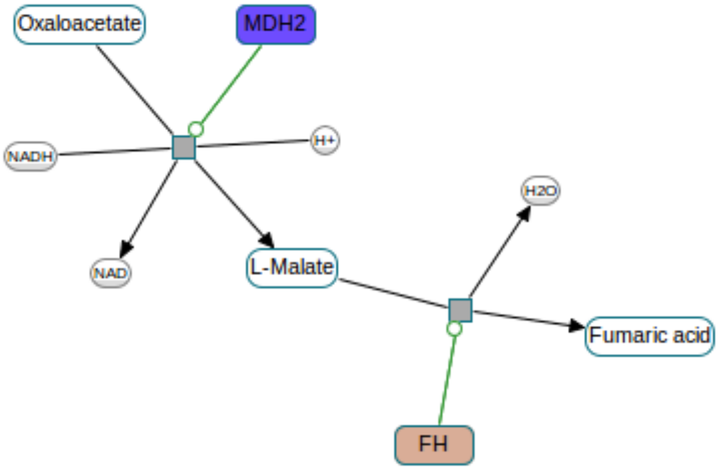

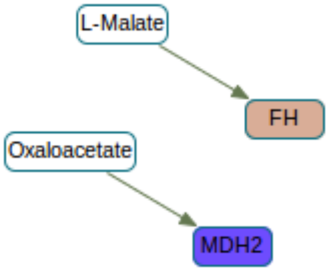

controls-production-of
This is a directed relation from a protein to a small molecule. The protein is controlling the production of the small molecule. The reaction is supposed to change the concentration of the small molecule. The produced small molecule (output) cannot also be an input. This relation can be useful for studying metabolic events.

Example: Consider same reactions above with MDH2 and FH. This pattern produces below interactions.

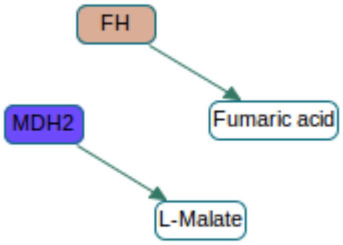

When consumption-controlled-by and controls-production-of used at the same time, we get a graph that can show the flow of the metabolic events as shown below. But users should be aware that the proteins may not be always promoting the flow, but can also be inhibiting it.

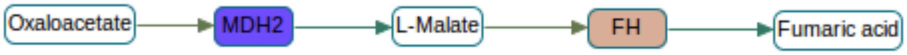

controls-transport-of-chemical
Directed relation from a protein to a non-generic small molecule, where the protein controls a reaction that changes the cellular location of the small molecule. This relation implies the concentration of the small molecule changes in at least one cellular location.

Example: SAR1B controls transportation of a Complex of APO proteins bound with cholesterols, triglycerides, phospholipids, and cholesterol esters.

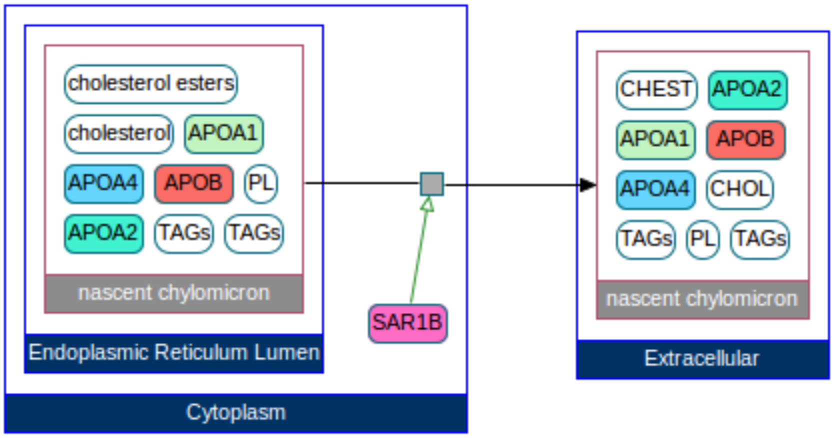

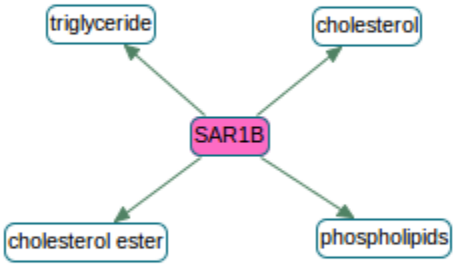

chemical-affects

This is a directed relation from a small molecule to a protein. The small molecule shows signs of affecting either activity or the state of the protein. This relation is essential for showing drugs in networks.

through binding

Similar to in-complex-with pattern, but this one is directed and it is from the small molecule to the protein.

<u>Example:</u> Testosterone and DHT affects activity of AR, SRC and PELP1 through binding.

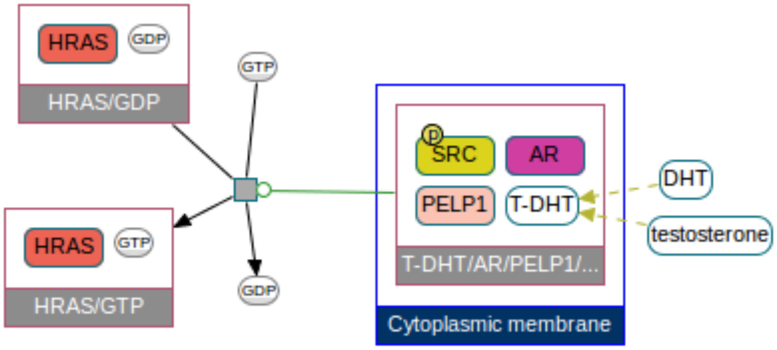

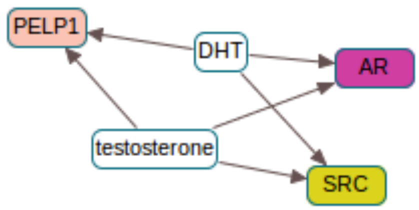

through a control

A small molecule is controlling an interaction and a protein is a participant of this interaction.

Example: Testosterone and DHT affects state of HRAS by controlling a reaction that modifies it (refer to the same BioPAX graph above).

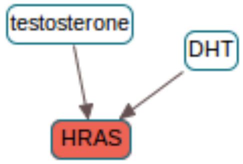

reacts-with

This is an undirected relation between two small molecules that are substrates to the same biochemical reaction. None of the molecules are also in products.

Example: UDPglucuronate reacts with Phenol to produce O-glucuronide and UDP.

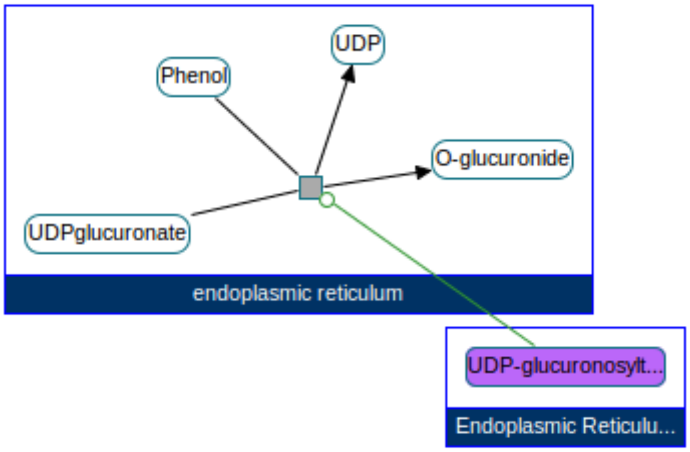

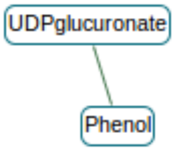

used-to-produce

This is a directed relation between small molecules. First one is a substrate of a biochemical reaction that produces the second molecule. Both molecules are only on one side of the reaction.

Example: Refer to the same example above where UPDglucuronate and Phenol is used to produce O-glucuronide and UDP.

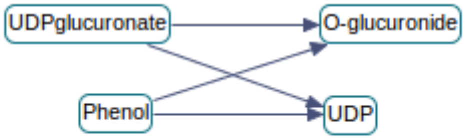

Example pathways using binary interactions

In this section, we try to redraw some popular pathway diagrams using binary interactions and Pathway Commons data. To produce these binary network, we execute a paths-between query in the large binary network derived from Pathway Commons, using one or two of the interaction types.

The Citric Acid Cycle

TCA cycle from wikipathways

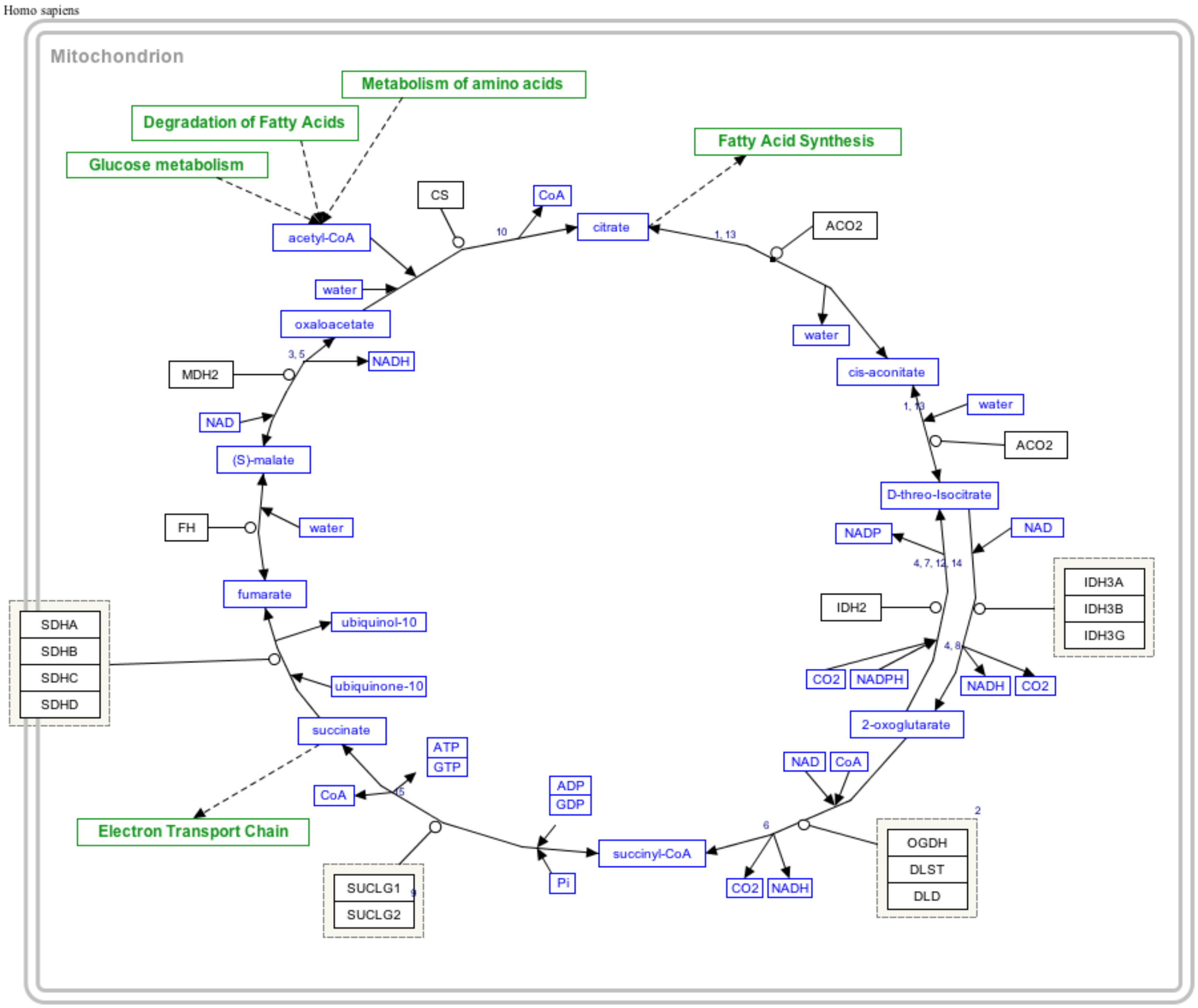

Binary network from Pathway Commons

With catalysis-precedes and in-complex-with interactions:

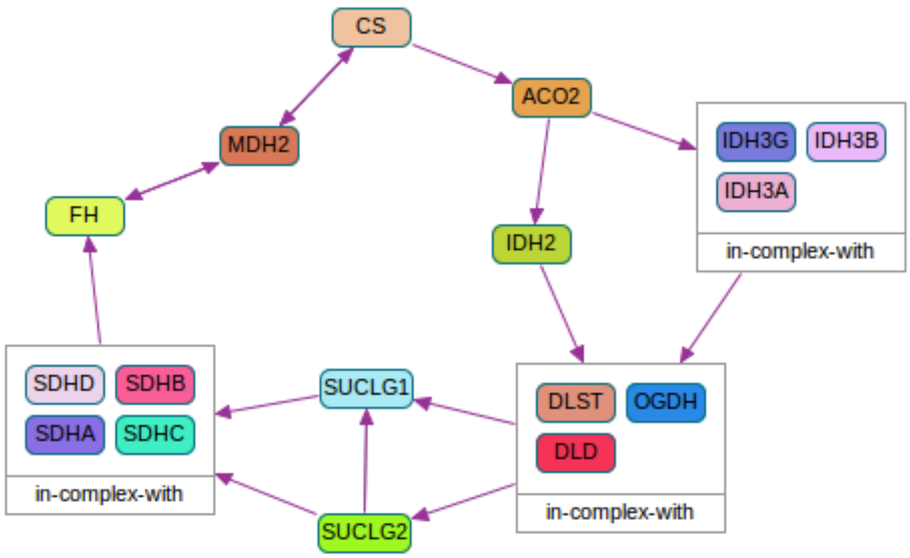

With consumption-controlled-by and controls-production-of interactions:

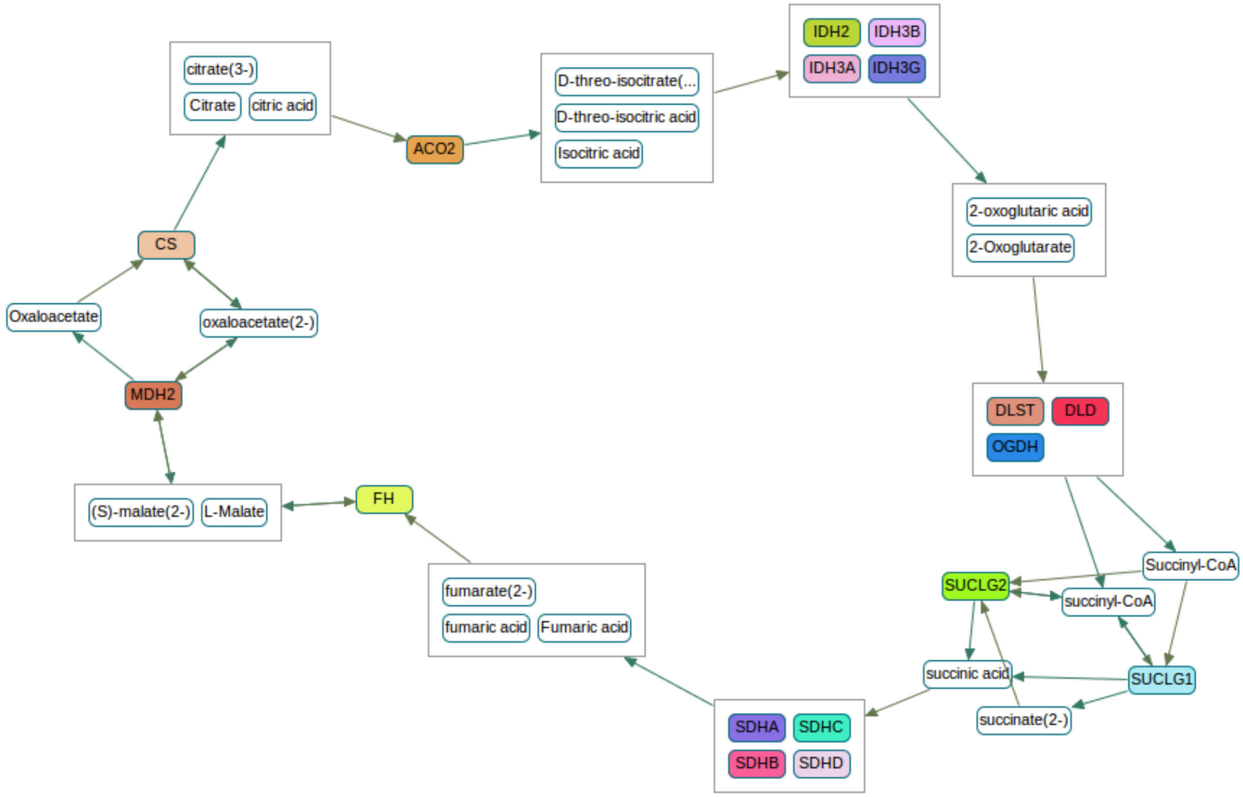

BMP signaling

Wiki pathways

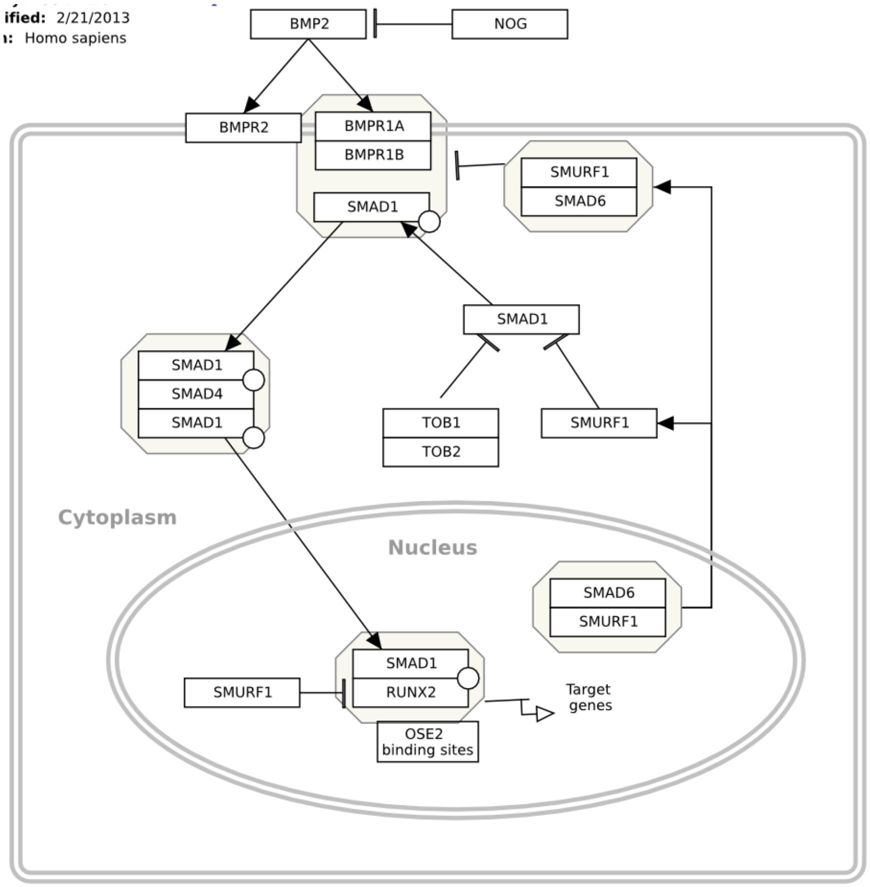

Binary network from Pathway Commons

With controls-state-change-of and controls-degradation-of interactions:

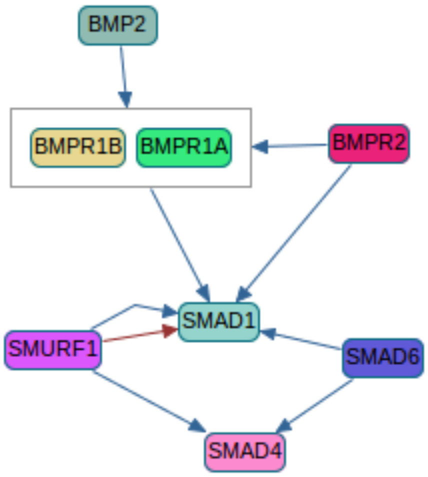

AMPK signaling

Wiki pathways

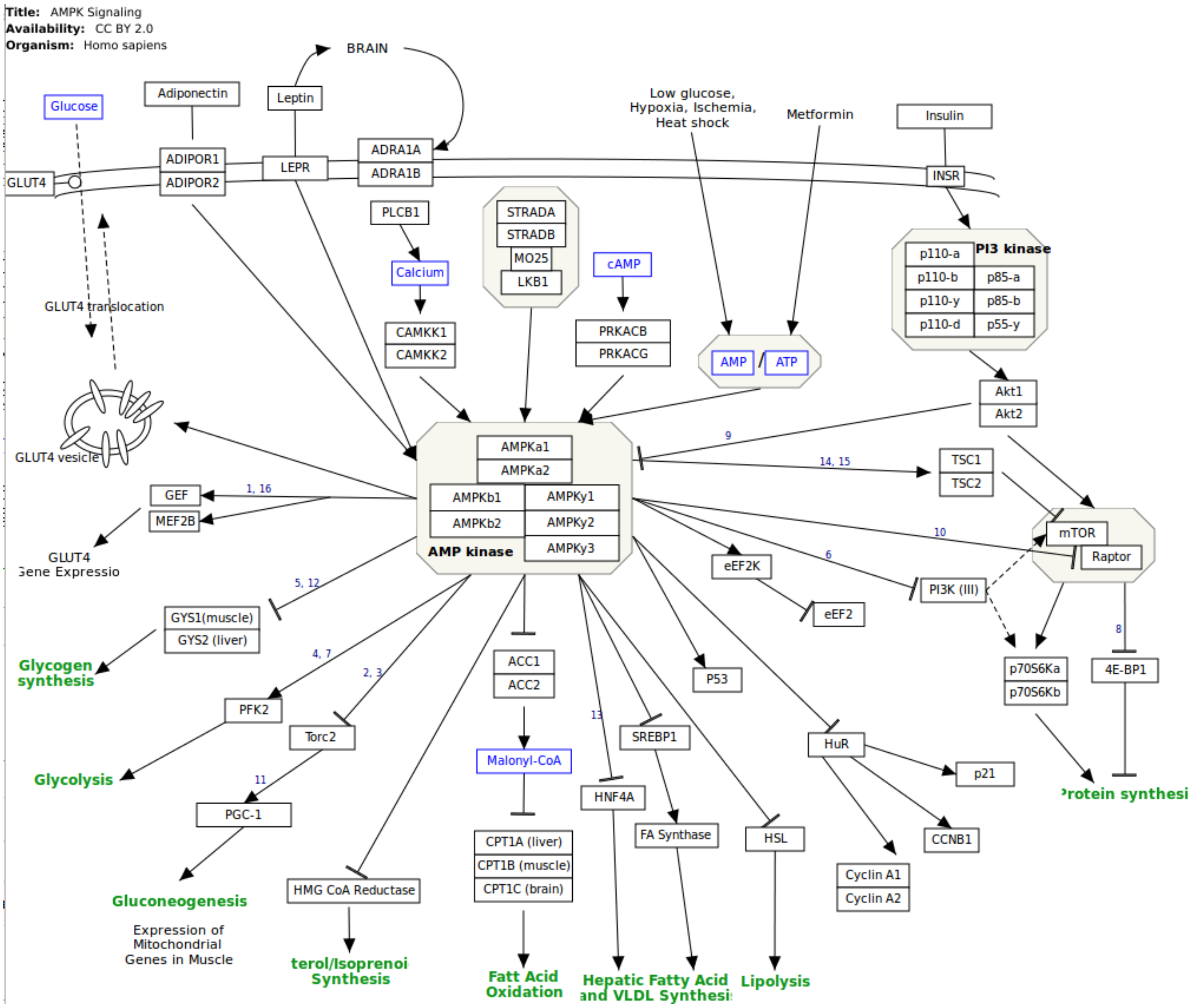

Binary network from Pathway Commons

With controls-state-change-of and controls-expression-of interactions (AMPK proteins are the ones starting with PRKA):

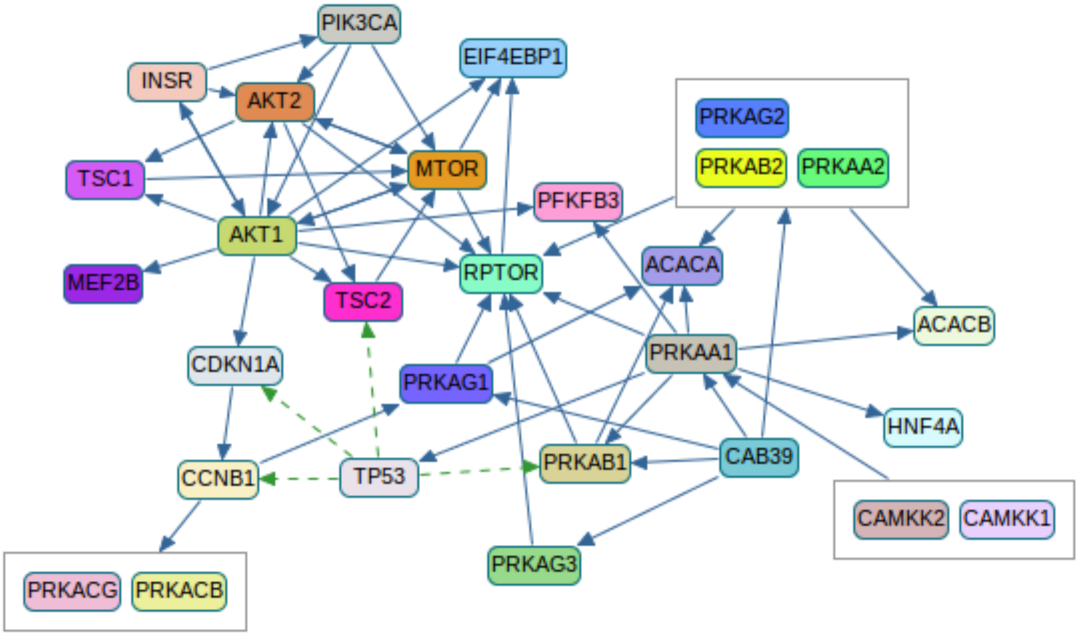

With interacts-with interactions:

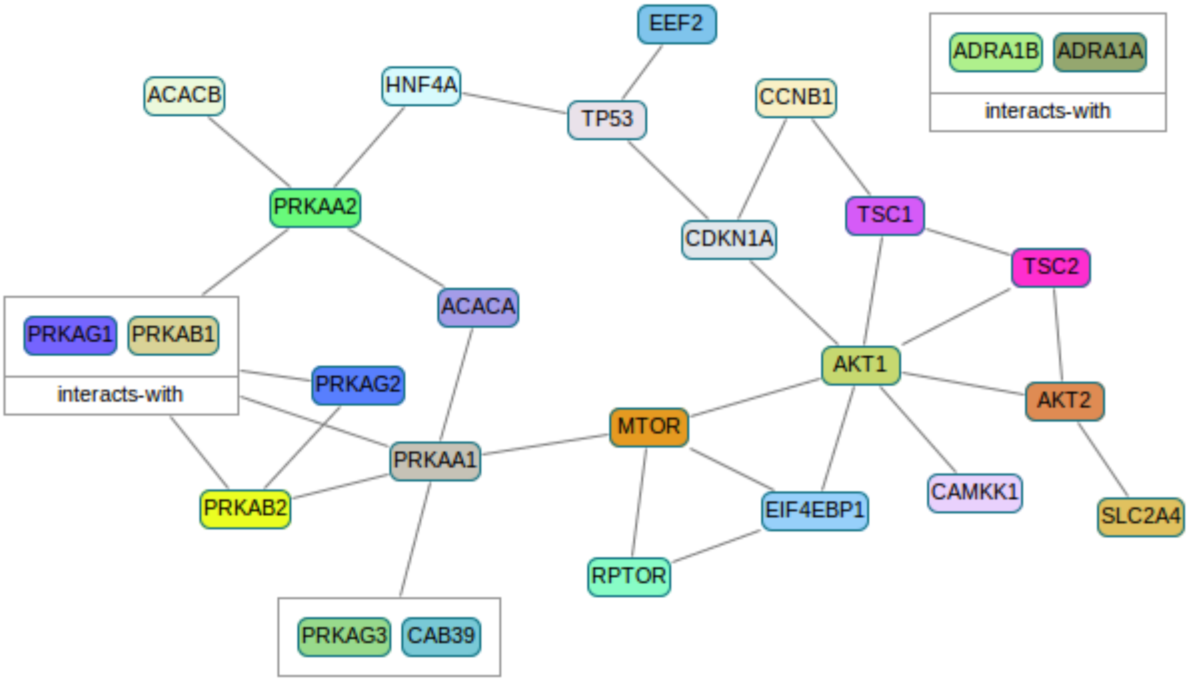

ABC-mediated transport

Reactome

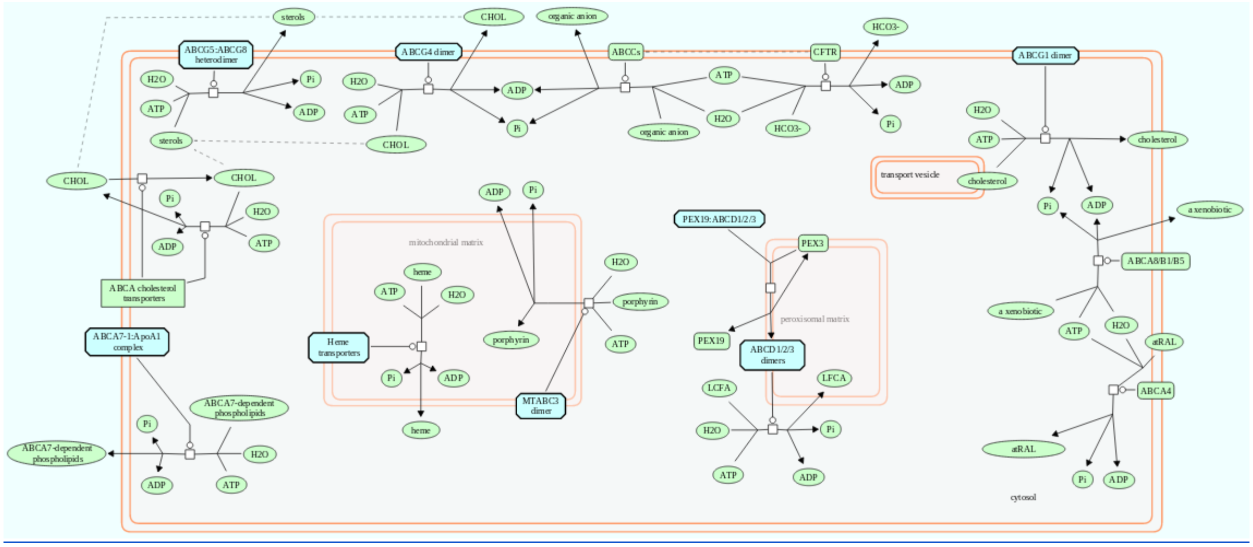

Binary network from Pathway Commons

With controls-transport-of-chemical interactions:

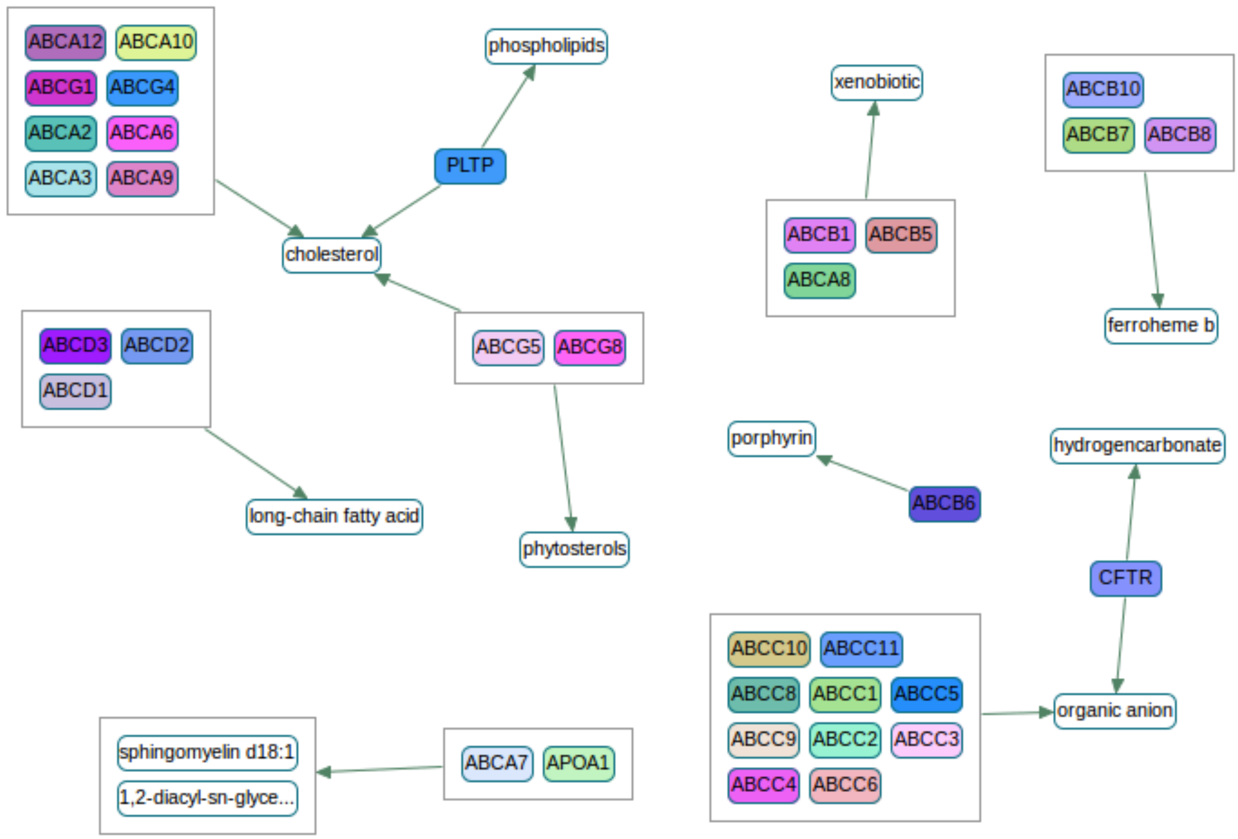

Supplement: gkz946_Supplemental_File [file gkz946_supplemental_file.pdf]
